# Rare Case of Double Esophageal Web in a Two-Year-Old

**DOI:** 10.7759/cureus.57784

**Published:** 2024-04-07

**Authors:** Archana Reddy Bongurala, Ayesha Fatima, Rahul Kashyap

**Affiliations:** 1 Pediatrics, Omni Family Health California, Bakersfield, USA; 2 Gastroenterology, Beaumont Health, Royal Oak, USA; 3 Medicine, Drexel University College of Medicine, Philadelphia, USA; 4 Global Clinical Scholars Research Training (GCSRT), Harvard Medical School, Boston, USA; 5 Research, Global Remote Research Program, St Paul, USA; 6 Critical Care Medicine, Mayo Clinic, Rochester, USA

**Keywords:** choking, endoscopic dilation, pediatrics, stenosis, esophageal web

## Abstract

Congenital esophageal stenosis (CES) is an uncommon condition that poses diagnostic and therapeutic challenges due to its rarity and clinical presentation similar to other esophageal disorders. Symptoms typically start with dysphagia around the introduction of solid foods.

A broad range of potential differential diagnoses contributes to a delay in obtaining a definitive diagnosis and administering the proper treatment.

We report a two-year-old boy who presented with difficulty swallowing solid foods since 11 months of age, manifesting as choking and gagging. Initial evaluation revealed a double esophageal web, with proximal stenosis detected in an esophagram. Despite two endoscopic dilations and cauterization of the proximal web, a second web in the middle third of the esophagus was found. Subsequent dilatation successfully improved symptoms, and the child began tolerating table foods. This case report aims to contribute to the limited existing literature on CES and to add to the clinical practice in the diagnosis and treatment of this uncommon congenital anomaly.

## Introduction

Congenital esophageal stenosis (CES) is extremely rare in infants and children, affecting one in every 25000 - 50000 live births with slight male predominance [1,2]. CES, frequently observed in pediatric individuals, can be characterized according to pathohistological discoveries. The common manifestations include tracheobronchial remnants (TBR, the most prevalent), fibromuscular thickening leading to stenosis, and the presence of esophageal web [1]. Tracheobronchial remnants are a type of congenital esophageal stenosis characterized by the abnormal persistence of embryonic respiratory tissue (tracheal origin) within the esophageal wall [3]. These misplaced remnants contribute to narrowing of the esophagus, hindering proper swallowing [3].

Esophageal stenosis is a rare condition in children and requires good clinical acumen for diagnosis. Infants are usually asymptomatic until the introduction of solid food when they develop dysphagia or recurrent vomiting and aspiration. Prompt diagnosis is important to obviate the need for major surgery. Minimally invasive endoscopic techniques and surgical intervention are the cornerstones of management for CES [4]. We report a rare case of double esophageal web in a two-year-old boy.

This article was previously presented as a poster at the CONF2019 - The Annual Meeting of NASPGHAN, APGNN & CPNP on October 17, 2019.

## Case presentation

A two-year-old boy with no significant past medical history presented to our Gastroenterology Outpatient Clinic with difficulty swallowing solid foods starting at 11 months of age. He tolerated pureed foods well and started having symptoms of choking and gagging after introduction of table foods. He was seen by occupational therapy a month prior to presentation for feeding difficulties when he was given oral exercises which did not help. No complaints of abdominal pain, constipation, diarrhea, blood in stool and no weight loss were noted. He has been otherwise growing and developing well. During the initial assessment, his vital signs were all within normal limits, including a normal body temperature, normal blood pressure, age-appropriate heart rate and respiratory rate, and good oxygen saturation. Physical examination was normal. Laboratory evaluation showed no evidence of metabolic acidosis. Abdominal radiographs showed a non-obstructive bowel gas pattern.

**Figure 1:**
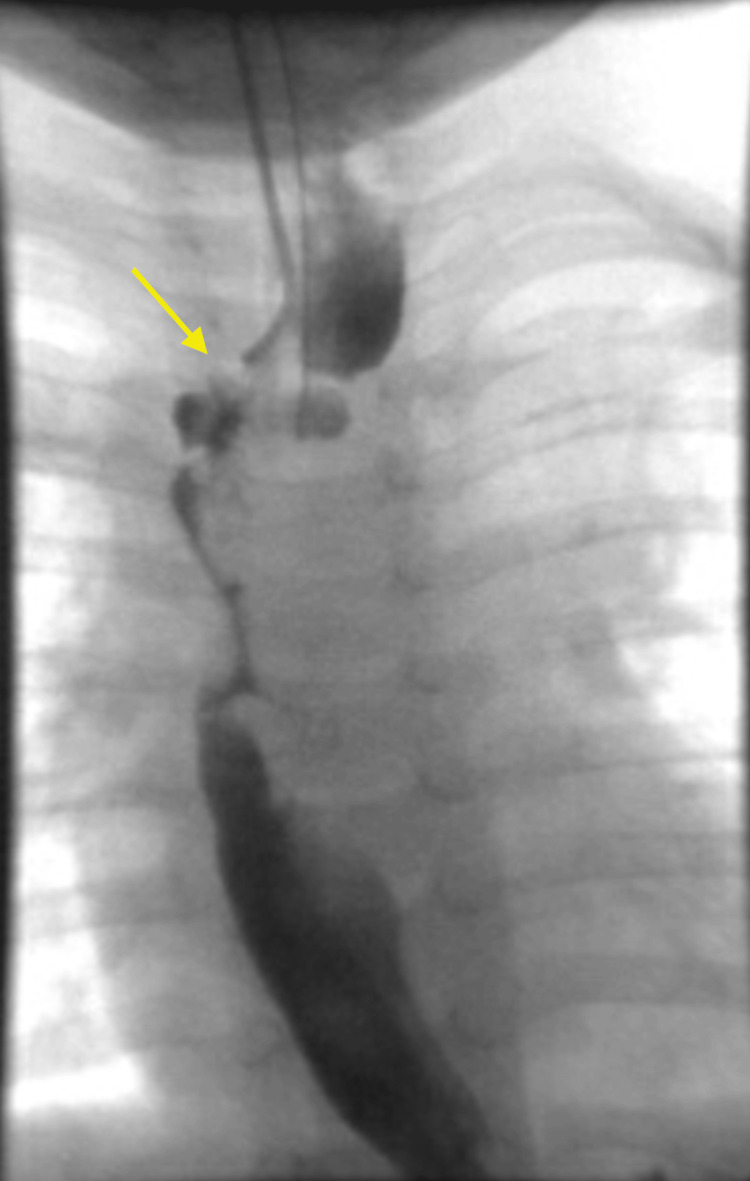
Kink/narrowing in the region of the proximal esophagus at the level of the thoracic inlet

**Figure 2:**
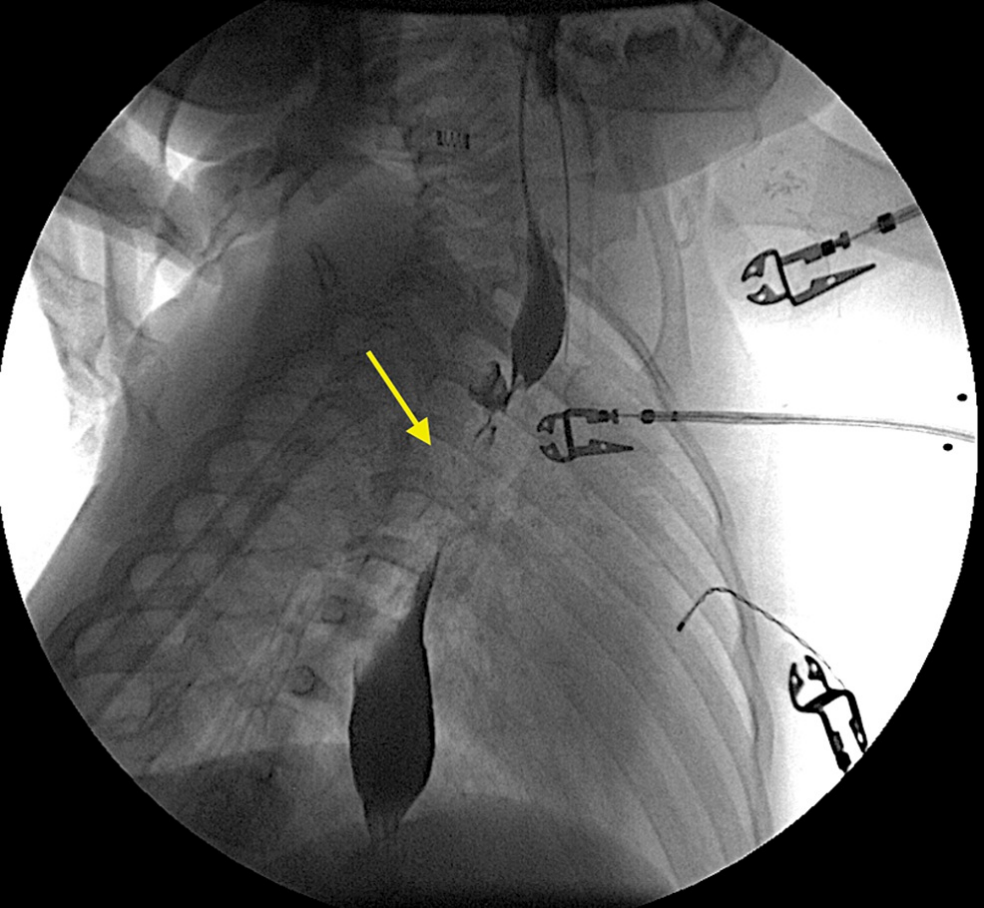
Catheter-directed esophagram demonstrating what appears to be an approximately 2-3mm thick and 8-9mm long esophageal web dividing the proximal third of the esophagus into two lumens.

**Figure 3:**
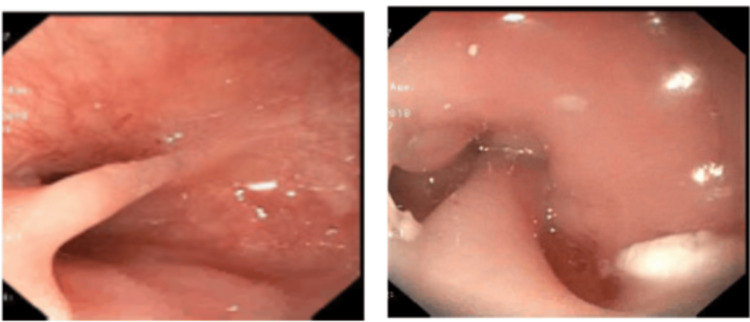
Endoscopy showing ring at 10cm and web at 15cm on endoscopic examination

An initial catheter-directed esophagram (Figures 1, 2) demonstrated proximal esophageal stenosis. Two courses of endoscopic dilations did not improve symptoms. Chest CT showed no evidence of a vascular ring. Pediatric surgeon was involved who cauterized the proximal web at 10 cm (Figure 3) but found difficulty in advancing the scope further down. A second esophageal web in the middle third of the esophagus at 15 cm (Figure 3) was discovered which was severed on a later esophagogastroduodenoscopy (EGD). Subsequent dilatation improved his symptoms significantly. He started tolerating table foods. He has had no further complaints of choking or difficulty swallowing solid foods. On follow-up at six months post procedure, the child continued to tolerate solid food well with no evidence of vomiting or choking and continued to gain adequate weight gain.

## Discussion

Congenital esophageal web is a thin membrane of tissue causing partial or complete luminal obstruction and is a rare cause of dysphagia in children. Feeding and swallowing disorders in children are considered a major challenge owing to a wide differential diagnosis. There can be a number of causes for dysphagia, including structural deficits, neurological conditions, medical, genetic, metabolic, and degenerative diseases. Among anatomical abnormalities, esophageal stricture is reported to be a rare but possible cause of dysphagia. Esophageal stenosis can be congenital or acquired. Congenital esophageal stenosis may exist in one of the following three forms: fibromuscular stenosis, esophageal membrane or web, and tracheobronchial remnant [1,2]. Acquired stenosis could be from traumatic, inflammatory, peptic, or post-surgery [1]. 

Congenital esophageal stenosis rarely presents in the neonatal period because the onset of symptoms usually begins with introduction of solid foods around four to 10 months of age [2]. Maintaining a high level of suspicion, obtaining a precise medical history, and conducting an esophagogram are crucial elements in reaching a diagnosis [2]. Endoscopy plays a crucial role in detecting stenosis, excluding esophagitis and foreign bodies, and facilitating biopsy whenever feasible. pH monitoring and manometry can also be valuable diagnostic tools [2]. Intraesophageal ultrasonography is helpful both for diagnosing CES and to distinguish tracheobronchial heterotopia [5]. In the treatment of CES, surgery and endoscopic approaches are crucial. While endoscopic interventions offer a potentially effective and minimally invasive option, it is essential to carefully weigh the risks associated with therapies and therapeutic margins [6].

In our case the infant tolerated breastfeeds/formula and pureed food well initially and symptoms started once table foods were introduced at around 11 months. This gave us a suspicion of an obstructive disorder. The workup began with an upper GI with barium swallow which showed narrowing at the level of proximal esophagus. Repeated attempts of balloon dilation were unsuccessful. An interesting and rare finding that was seen in our case was that once the proximal web was cauterized using hot forceps and when tried to advance the catheter further, a second web was discovered in the middle third of the esophagus. The patient was scheduled for a repeat endoscopy to cauterize the second web followed by balloon dilatation. The patient’s symptoms improved significantly after this and the patient was able to tolerate solid foods well. Although previous studies have documented successful treatment of single esophageal webs in children using endoscopic dilation [7,8], this case report presents a unique finding: two esophageal webs in a child, effectively treated with a combination of electrocauterization and balloon dilation.

## Conclusions

Congenital esophageal stenosis is a rare cause of dysphagia in children and symptoms don’t usually present until the introduction of solid foods. It can have varied presentations. Barium esophagogram is the initial and most important tool in diagnosing esophageal stenosis. Endoscopic balloon dilation is the primary treatment and is successful in most cases while some might require electrocauterization. Children who present with difficulty feeding might have underlying medical diagnoses that might need endoscopic intervention and treatment. Early referral to a pediatric Gastroenterologist might be helpful in such cases.
